# Exploring the experiences of loneliness in adults with mental health problems: A participatory qualitative interview study

**DOI:** 10.1371/journal.pone.0280946

**Published:** 2023-03-07

**Authors:** Mary Birken, Beverley Chipp, Prisha Shah, Rachel Rowan Olive, Patrick Nyikavaranda, Jackie Hardy, Anjie Chhapia, Nick Barber, Stephen Lee, Eiluned Pearce, Brynmor Lloyd-Evans, Rosie Perkins, David McDaid, Theodora Stefanidou, Roz Shafran, Alexandra Pitman, Sonia Johnson

**Affiliations:** 1 Division of Psychiatry, University College London, London, United Kingdom; 2 The Loneliness and Social Isolation in Mental Health Research Network Co-Production Group, Division of Psychiatry, University College London, London, United Kingdom; 3 NIHR Mental Health Policy Research Unit COVID-19 Co-Production Group, Division of Psychiatry, University College London, London, United Kingdom; 4 Centre for Performance Science, Royal College of Music, London, United Kingdom; 5 Faculty of Medicine, Imperial College London, London, United Kingdom; 6 Care Policy and Evaluation Centre, Department of Health Policy, London School of Economics and Political Science, London, United Kingdom; 7 Great Ormond Street Institute of Child Health, University College London, London, United Kingdom; 8 Camden and Islington NHS Foundation Trust, London, United Kingdom; Universita degli Studi di Milano-Bicocca, ITALY

## Abstract

**Background:**

Loneliness is associated with many mental health conditions, as both a potential causal and an exacerbating factor. Richer evidence about how people with mental health problems experience loneliness, and about what makes it more or less severe, is needed to underpin research on strategies to help address loneliness.

**Methods:**

Our aim was to explore experiences of loneliness, as well as what helps address it, among a diverse sample of adults living with mental health problems in the UK. We recruited purposively via online networks and community organisations, with most interviews conducted during the COVID-19 pandemic. Qualitative semi-structured interviews were conducted with 59 consenting participants face-to-face, by video call or telephone. Researchers with relevant lived experience were involved at all stages, including design, data collection, analysis and writing up of results.

**Findings:**

Analysis led to identification of four overarching themes: 1. What the word “lonely” meant to participants, 2. Connections between loneliness and mental health, 3. Contributory factors to continuing loneliness, 4. Ways of reducing loneliness. Central aspects of loneliness were lack of meaningful connections with others and lack of a sense of belonging to valued groups and communities. Some drivers of loneliness, such as losses and transitions, were universal, but specific links were also made between living with mental health problems and being lonely. These included direct effects of mental health symptoms, the need to withdraw to cope with mental health problems, and impacts of stigma and poverty.

**Conclusions:**

The multiplicity of contributors to loneliness that we identified, and of potential strategies for reducing it, suggest that a variety of approaches are relevant to reducing loneliness among people with mental health problems, including peer support and supported self-help, psychological and social interventions, and strategies to facilitate change at community and societal levels. The views and experiences of adults living with mental health problems are a rich source for understanding why loneliness is frequent in this context and what may address it. Co-produced approaches to developing and testing approaches to loneliness interventions can draw on this experiential knowledge.

## Introduction

Inter-relationships between loneliness and mental health problems are the focus of a growing body of literature [1]. Loneliness is defined as a subjective experience where individuals feel there is a discrepancy between social relationships that they desire to have and those they actually have [2]. Loneliness is more prevalent among people with mental health problems than the general population [1, 3, 4].

Important associations are found between loneliness and a range of health indicators. It is a risk factor for multiple poor physical health outcomes, including early mortality, impaired cognition, hypertension, stroke, and cardiovascular disease [5–8]. Health service use is greater among lonely people, especially older people [9, 10]. Regarding mental health, loneliness appears to put people at risk of onset of depression [11, 12], whilst loneliness (and a closely related construct, lack of subjective social support) is associated longitudinally with recovering less well from mental health problems [13, 14].

Whilst the evidence linking loneliness to mental ill health, as well as its inherently distressing nature, make loneliness a potentially promising focus for developing strategies to improve quality of life and outcomes among people living with mental health problems [15], few evidence-based and implementation-ready interventions are available [16]. Strategies to reduce loneliness are more likely to be successful if rooted in an understanding of what people with mental health problems mean when they say they are lonely, how this relates to experiences of mental distress, and what they find improves or exacerbates loneliness. Much of the empirical research on loneliness and mental health has deployed measures that treat loneliness as a straightforward uni-dimensional phenomenon. This is despite philosophical, historical and experiential writing suggesting that the term captures a complex cluster of emotions and experiences [17, 18]. Currently used measures are also not tailored to investigating loneliness in the context of mental ill-health, nor have they been developed in collaboration with people with relevant lived experience.

Qualitative research on lived experiences of loneliness among people with mental health problems has important potential to yield a deeper account of the nature of such experiences and what improves or exacerbates loneliness. Such an understanding should underpin further research, including development of interventions, measures and hypotheses for quantitative investigations. The few published investigations are small-scale, and suggest a complex, intertwined relationship. A phenomenological study of people diagnosed with “borderline personality disorder” [19] found that participants perceived loneliness as rooted in traumatic early experiences and strongly associated with negative feelings about self and others. Participants also described feeling disconnected from those around them and on the outside of social activities at which they were present. Lindgren and colleagues [20] interviewed five individuals with mental health problems, who described multifaceted and shifting experiences of loneliness that varied with life situation but were also persistent. A meta-synthesis of studies on the experience of loneliness among young people with depression identified a range of factors, including depressive symptoms, non-disclosure of depression, and fear of stigma, which perpetuated cycles of loneliness and depression [21]. However, the qualitative literature on loneliness experiences among people living with mental health problems overall remains very limited in scope and size.

Our aim in the current study is therefore to develop an understanding of the lived experiences of loneliness among a broad range of people living with mental health problems. This was identified as a high priority evidence gap by the UKRI (United Kingdom Research and Innovation) Loneliness and Social Isolation in Mental Health Research Network, a cross-disciplinary research network established to advance research on the relationship between loneliness and mental health [22].

## Methods

We present data from a qualitative interview study [23] which employed a co-production approach [24], involving collaboration between people with relevant lived experience, clinicians and university-employed researchers (some of the team had multiple relevant perspectives). Semi-structured individual interviews were used to explore the experiences of loneliness in adults with mental health problems.

Ethical approval was obtained from the University College London Research Ethics Committee on 19/12/2019 (Ref: 15249/001), with a subsequent amendment approved to extend the study to include experience of the COVID-19 pandemic among people with mental health problems, meeting a pressing need for this. In this paper we report only on findings relevant to the original question regarding experiences of loneliness and their relationship with mental health. Findings relevant to the pandemic are reported in three other papers [23, 25, 26].

### Research team

A team of Lived Experience Researchers, (LERs), drawing on their own experiential knowledge about living with mental health problems, and other researchers from the UKRI Loneliness and Social Isolation and Mental Health network and the National Institute for Health and Care Research Mental Health Policy Research Unit (MHPRU) planned and conducted the study. The team included clinical academics and non-clinical researchers from a range of backgrounds (including qualitative and mixed methods research, health policy, health economics, and the arts). The research team met weekly by Zoom video call [27] to plan the study and discuss progress. Most interviews were conducted by thirteen LERs involved in the study, except for eight telephone interviews conducted by MB, an experienced qualitative researcher and occupational therapist. Three LERs were employed in university research roles; others had honorary research contracts with University College London. Eleven of the LER interviewers were female and five were from minority ethnic backgrounds. The LERs received training on conducting face-to-face and online interviews and obtaining written and verbal informed consent. A weekly lived experience reflective space provided LERs with emotional support and space to discuss the research process and emotional impact, peer-facilitated by four experienced LERs.

### Sampling and recruitment

Purposive sampling was used to ensure diversity regarding participants’ diagnoses, use of mental health services, and demographic characteristics, including age, gender, ethnicity, and sexuality, and whether they lived in rural or urban areas. We reviewed our sample during recruitment and implemented targeted strategies to ensure diversity. These included approaching community organisations working with Black and Minority Ethnic communities and using targeted recruitment materials.

Participants were eligible to take part if they were aged 18 years or over, had a self-reported mental health problem and lived in the UK. We recruited through three London-based community organisations (a mental health charity, a homeless charity and a community arts organisation), as well as through social media, especially Twitter, supported by the Mental Elf. Several charities and community organisations supporting people with mental health problems also agreed to disseminate an invitation to participate to their networks. Potential participants contacted the research team by email. Researchers then checked eligibility, provided a participant information sheet, answered questions about the study, and booked interviews.

### Data collection

The topic guide (see S1 Appendix) was developed collaboratively by members of the Loneliness Network’s Co-Production Group to explore the nature of loneliness experiences, their relationship to mental health, and alleviating and exacerbating factors. Prompts were included for questions asked to ensure topics were fully explored. Following the onset of the COVID-19 pandemic, further questions were added regarding experiences of living with mental health problems during the pandemic (see S2 Appendix for revised topic guide). Semi-structured interviews took place between March and July 2020. Ten participants were interviewed before the introduction of COVID-19 infection control measures, (two face-to-face with LERs and 8 by phone with MB and 49 participated in online interviews with LERs following the pandemic’s onset. Informed consent (verbal or written) was obtained prior to all interviews. All interviews were audio-recorded, with verbatim transcripts produced by an external transcription company. All transcripts were then checked by the researchers and any identifying information was anonymised.

### Analysis

We took a participatory approach to the analysis as a large team of researchers from varying backgrounds. We used Template Analysis [28], a form of thematic analysis [29] involving a codebook approach. Analysis involved defining and organising themes using a coding template, which was developed and refined during data analysis through an iterative approach. All interview transcripts were analysed by four Lived Experience Researchers (RRO, BC, PS and PN) and a network researcher (MB) facilitated by NVivo 11 software [30]. MB undertook a preliminary analysis of three transcripts, reading and re-reading to identify initial themes. Three LERs independently analysed one each of those transcripts and discussed points of divergence. Differences were compared with MBs initial themes identified to highlight areas requiring closer examination and to ensure the coding captured complex and nuanced data relevant to the research topic. The aim was to capture richer data, to guide further coding, and not to seek a consensus on meaning, in keeping with the approach of reflexive thematic analysis [29]. All themes formed an initial coding template. A further set of five transcripts were then analysed individually and the further initial themes were then discussed between the researchers conducting the analysis. This group reflected on the themes together to ensure important ideas in the transcript had not been overlooked, and to refine the initial coding template.

The analysis followed an iterative process where the core coding team met fortnightly with the wider research team to discuss and refine the developing list of themes in the coding template, obtaining further perspectives on the analysis. All transcripts were then coded following the final template.

## Results

Fifty-nine participants were recruited. The majority were female (n = 41, 69%), aged between 25–54, and living in a city (n = 43, 73%). The main ethnic groups reported were White British (n = 32, 54.2%), White Other (n = 8, 13.5%) and Black/Black British (n = 7, 11.9%). Table 1 outlines the demographics of all 59 participants. Our analysis identified four over-arching themes.

**Table 1:** Demographics of 59 participants.

**Gender**	
Female	41 (69%)
Male	18 (31%)
Other categories	None
**Age**	
18–24	2 (3.4%)
25–34	15 (25.4%)
35–44	15 (25.4%)
45–54	16 (27.1%)
55–69	5 (8.5%)
≥ 70	4 (6.8%)
No information available	2 (3.4%)
**Ethnicity**	
White British	32 (54.2%)
White Other	8 (13.5%)
Mixed/multiple ethnic groups	5 (8.5%)
Asian/Asian British	6 (10.2%)
Black/Black British	7 (11.9%)
Other Ethnic Group	1 (1.7%)
**Sexual orientation**	
Lesbian/gay/bisexual/other non-heterosexual^a^	9 (15.3%)
Heterosexual	43 (72.9%)
Prefer not to answer or information not available	7 (11.8%)
**Self-reported diagnosis**	
Mood disorders (depression, anxiety, PTSD)	26 (44%)
Personality disorder	7 (12%)
Bipolar disorder	8 (13.5%)
Psychosis/schizophrenia/	7 (12%)
Other (addictions, suicidal thoughts, OCD)	6 (10%)
Not stated	11 (18.5%)
**Current/past use of mental health services**	
None (n = 13), or on waiting list (n = 3)	16 (27.1%)
Inpatient services^b^	3 (5.1%)
NHS community mental health services^c^	28 (47.4%)
GP or primary care counselling	5 (8.5%)
Private sector psychotherapy only	2 (3.4%)
Voluntary sector mental health services only	4 (6.8%)
No information available	1 (1.7%)
**Urban/Rural location**	
City	43 (73%)
Town	12 (20.3%)
Village	3 (5%)
No Information available	1 (1.7%)

^a^ Lesbian/gay/bisexual/other non-heterosexual included: gay (n = 1), lesbian (n = 1), bisexual (n = 6), pansexual (n = 1)

^b^Inpatient services included: acute inpatient ward (n = 2), crisis house (n = 1)

^c^Community mental health services included: community mental health team (n = 22), reablement team (n = 1). therapist (n = 3), NHS peer support service (n = 2)

### Theme one: What the word “lonely” meant to participants

Loneliness was described in a variety of ways, including as an emotional or physical state, or through accounts of lack of connection, feeling of belonging, or love and support. A unifying element was that it was not just an unwanted lack of contact with people but had important psychological elements.

#### 1.1 How loneliness feels emotionally

Participants often used words synonymous with low mood to describe emotional aspects of loneliness, including “*sad*”, “*depressing*”, “*miserable*”, “*despondent*” and “*tearful*”, and generally conveyed that loneliness was a painful state negatively impacting mental health.

#### 1.2 Not feeling connected

Being alone featured in definitions, but the experience of not feeling connected to others was more prominent:

“*The last 35 years has been lonely*…*I mean I’ve got a husband*, *I’ve got four kids and everything else*, *but it doesn’t stop you from feeling lonely*.”

[P47, female, White British, 46–55, urban]

People spoke about emotional, psychological and spiritual disconnection and “*not sharing energy*”. They often described being in the company of others, yet feeling unable to connect with them emotionally. This was a chronic state for some, or coincided with periods of mental unwellness for others:

*"Remember becoming very depressed and*…*with a group of friends doing things I used to enjoy and literally not feel any connection with the people around me*, *especially if I was having a lot of intrusive thoughts*…*"*

[P44, female, White British, 36–45, urban]

Most interviews occurred under COVID lockdown when many social and mental health communications were digital. Physical presence clearly mattered to many people. One participant described loneliness as:

“*Less connection to people and specifically decreasing face-to-face contact*.”

[P28, female, White British, 56–65, urban]

Loneliness was sometimes conceptualised as a feeling of disconnection going beyond relationships merely with people:

“*Disconnect from the world*, *both the physical and the spiritual world as well*.”

[P37, female, Black, 26–35, urban]

Pets were mentioned as valued companions by several interviewees. Connections with them sometimes appeared on a par with humans, and their constancy was an asset that could be either cherished or missed.

“*Not having contact with other people or animals*” [P49, male, British Asian, 46–55, urban]

### 1.3 Lack of a sense of belonging

Some people described loneliness as related to objective isolation:

“*Some people*… *they have no choice*… *they don’t have the care and support from family and friends*.”

[P38, male, Black, 46–55, urban]

For others, loneliness involved not feeling they belonged in a social environment or community:

“*No*, *I don’t feel part of this community*, *not at all*…*I feel lonely where I live*, *yes*.”

[P51, female, White other, 46–55, urban]

“*Sometimes the feelings of*, *well I do belong to that community but do I really*? *Am I a bona fide member of it*? *Feel like I am a hanger-on in the community*.”

[P11, male, White British, 66–75, urban]

Some described a sense of thwarted belongingness, being ‘*on the outside looking in’* or ‘*missing someone or something*, *but not always knowing what*.*’*

Not being able to find others who shared values and interests was part of the sense of not belonging:

“*I feel like I struggle with my identity sometimes*, *like who am I and stuff like that*. *Yeah*, *I’m always like*, *trying to find like*, *my tribe*, *and like-minded people and stuff like that*.”

[P29, female, Black, 26–35, urban]

For one participant this feeling of being ‘other’ and not belonging followed relocation from a large city to a more rural area with a different recreational culture:

“*It’s not actually the act of being alone*, *it’s*… *not having [the right] people to do things with or to share things with*.”

[P2, female, 36–45, White British, urban]

Social class played a role in some people’s lack of a sense of belonging:

“*I’m from a working-class background*, *I’m very around middle-class professionals at work -who speak very differently to me*. *…I have to constantly think about how I’m coming across*, *how I’m speaking… makes me feel a bit disconnected…I rarely join them for lunch and things … I find it a bit like I’m an outsider*.”

[P37, female, Black African, 26–35, urban]

#### 1.4 Not feeling loved, supported or understood

Not feeling loved or supported underpinned many of the narratives, sometimes resulting in a deep sense of emotional pain. This applied whether participants were objectively isolated or not:

“*Don’t have anyone to speak to or anyone to support you*… *don’t have anyone to turn to*.”

[P34, female, Asian, 18–25, urban]

Lack of help and support in dealing with mental health problems was one source of such loneliness:

“*No one helps*, *no one cares*.”

[P4, female, White British, 26–35, urban]

Several participants recounted interpersonal rejections that had triggered self-doubts, and these ruminations undermined their mental wellbeing:

“*You think*…*this person is not interested in me*. *Then you start to*… *make some negative judgements about yourself*.”

[P10, male, Black British, 56–65, urban]

Not feeling understood was also described as increasing feelings of loneliness:

“*When I’m not feeling understood*… *it feels like I’m on my own little planet*.”

[P9, female, White other, 36–45, urban]

#### 1.5 Physical sensations of loneliness

A few participants described loneliness in terms of physical sensations, such as “*tightness or stabbing in the heart*”, and “*a body ache*”, but with most sensations relating to the digestive system, such as “*physically feel nauseous*”, “*hunger*”, “*craving*” or “*a blow in the guts*”.

### Theme two: Connections between loneliness and mental health

Participants described close connections between mental health and feelings of loneliness. These included loneliness leading to deterioration in mental health, and feeling lonely because of impacts of mental health problems.

#### 2.1 Loneliness causing or worsening mental health problems

Social isolation and loneliness may lead to negative thoughts, low mood and deteriorating mental health, and may prevent people accessing support from others that might help them stay well:

“*I think the sense of loneliness would have preceded the mental health problem*, *because then*… *if I had the social interaction then I suspect I would have probably managed to find some way to sort it out*.”

[P49, male, British Asian, 46–55, urban]

“*Loneliness can trigger depression in me*, *it can take me to a dark place*.”

[P59, male, White British, 46–55, urban]

“*The negative thoughts and the looping thoughts get much worse if I’m lonely*. *So*… *because seeing people helps interrupt them*, *without that*, *the anxious thoughts can get a bit out of control*”

[P15, demographic information not available]

#### 2.2 Impact of having long term mental ill-health on loneliness

Conversely, participants described how having an ongoing mental health problem contributed to erosion of social contacts and to loneliness:

“*My depression involved me sitting in the dark at night with the lights off*… *I used to be popular*. *People stopped coming to see me because I’d changed*. *Yeah*, *so I became lonely then in the end*.”

[P10, male, Black British, 56–65, urban]

Ultimately this participant felt there was no choice but to be on their own and anxiety reduced their ability to reach out to people.

Some participants found managing day-to-day life tasks, such as handling household chores, in addition to coping with their mental health problems challenging, resulting in less time available to address their loneliness:

“*Always playing catch up to get everything sorted in my life*… *I have to find more ways to either quickly execute the things that need to be done to run a life and a flat or be very strict about just cutting things that don’t seem to matter so much and so concentrate more on*… *finding ways to sort of overcome loneliness*.”

[P28, female, White British, 56–65, urban]

#### 2.3 Cyclical relationship

Participants described a cyclical relationship between feeling lonely and changes in their mental health:

“*It’s a bit of a vicious cycle because I think feeling lonely…will make it worse*, *you need to be able to talk to people*… *So*, *it’s a bit of a cycle*… *your mental health means you can’t connect to people*, *then not being able to connect makes your mental health worse and then you’re just cycling around*.”

[P46, female, white British, undisclosed, urban]

#### 2.4 Relationships between loneliness and specific mental health conditions and symptoms

Some participants identified links between particular mental health difficulties and loneliness, although many themes were found to be cross-cutting rather than condition-specific. Depressive symptoms and loneliness were often described by participants as reinforcing each other, as also described in some of the quotes above:

“*I’ve learnt from the depressive part of my disorder*, *that when I’m depressed I haven’t got the energy*, *and it’s*… *like you inhabit a different space to the one you normally do*. *It’s kind of like you’re* (behind) *a glass wall and you’re not able to connect* (emotionally) *with the person who’s on the other side of the glass*, *even if you wanted*.”

[P55, female, White British, 46–55, rural]

Impacts from anxiety, especially social anxiety, were also described, leading to difficulty in social situations and resulting loneliness:

“*So anxiety can make me feel*… *lonely even if I’m in a party*. *I can be in a party with all my friends*, *all just want me to be happy and free and relaxed and dance*, *enjoy the music*. *But I can’t come out of my shell*. *I’m constantly preoccupied with my anxiety and this sense of…not enjoying myself*. *…Then I do kind of feel very lonely*.”

[P8, male, White British, 36–45, urban]

“*If I’ve got nobody there who can sort of help fight against the social anxiety… the fear of the people around me combined with struggling to start up conversations means I feel very lonely*.”

[P27, female, White British, 25–34, urban]

Participants also described loneliness resulting from or being reinforced by symptoms of psychosis:

“*When I first developed schizophrenia I felt very*, *very lonely*… *I didn’t know*,*… none of my friends has schizophrenia*. *I had constant voices giving me orders so ‘you’re not worthy sleeping’ or ‘you’re not worthy’ of sitting down*. *I’d just stand there in a room*.”

[P8, male, White British, 36–45, urban]

“*Getting out the flat is hard because of mental health and then having conversations with friends*, *like the voices would tell me stuff like ‘they hate you’ ‘don’t talk to them’ so that side*. *And then sort of like lacking motivation sometimes to sort of hold conversation*.”

[P20, female, White British, 26–35, urban]

Others described loneliness as resulting from difficulties in forming relationships that they saw as part of their mental health condition: a participant who reported having a diagnosis of “borderline personality disorder” said:

“*Because of my condition*, *I worry about having relationships with people and that keeps me isolated and keeps me lonely*.”

[P1, female, White British, 36–45, urban].

#### 2.5 Stigma and social exclusion

A key factor reported by multiple participants was the negative impact of stigma related to mental health problems on relationships:

“*People walk away*. *You mention the word hospital*, *people walk*. *You mention the word psychiatry and people walk away*.”

[P50, male, White British, 56–65, urban]

“*I think because of what I have*, *and the stigma associated with personality disorders*… *in the press*,…*some of the family members*, *some work colleagues*, *I think if I’m feeling lonely then that sort*… *of stigma*, *I think it just all compounds more*”

[P30, female, White British, 36–45, urban]

Cultural taboos related to mental health were reported by some participants as having resulted not only in loneliness but in being ostracised:

“*People within my culture*… *my family don’t understand and friends from my culture don’t get it so I feel more isolated*. *I’m losing friends because of this*.”

[P34, female, Asian, 18–25, urban]

#### 2.6 Choice and control—Social withdrawal and masking as a coping mechanism

Some participants recognised an element of choice in not communicating with others or living in self-imposed isolation:

“*I could pick up the phone and ring someone anytime any day and I don’t…so that is sort of self-imposed isolation*.”

[P11, male, White British, 66–75, urban]

One participant, who had experienced childhood trauma said they avoided authentic connections, and always ‘*wore a mask*’ when relating to others because that kept them safe:

“*There’s certain parts of you*, *you just don’t let people in*. *And that can be lonely*.”

[P47, female, White British, 46–55, urban]

Choice and control, however, were complex, and beneath the conscious decisions there could be unconscious barriers or mental health factors:

“*It could be something which is self-inflicted for example*, *when I was depressed I felt lonely but some of it was that I felt comforted by being left alone and yet I also felt really anxious about being alone as well*.”

[P49, male, British Asian, 46–55, urban]

### Theme three: Factors contributing to ongoing loneliness

As well as impacts of mental health problems, participants identified contributing factors to loneliness that included both historic root causes and current triggers, which were internal and external in nature.

#### 3.1 Root causes of loneliness

Perceived internal root causes of loneliness included being an introvert, or having low self-esteem and self-confidence, and therefore spending less time around people:

“*Because I have issues of self-esteem and all that kind of thing*… *For example*, *I don’t go to birthdays and that kind of thing*. *Which causes me to feel lonely*.”

[P41, female, Black British, 26–35, urban]

Participants also identified earlier traumatic events such as bereavement, domestic violence and experiences of being bullied that negatively affected how they viewed other people and their social interactions. Thus, earlier external events such as trauma shaped ways of thinking and choices, resulting in current psychological underpinnings of loneliness:

“*Sometimes I feel comfortable to just go up to them* [at work function] *and interrupt and join in the conversation but other times I feel like ‘oh they haven’t included me therefore they don’t want me there’*…*and I think that has probably stemmed from you know being bullied at school*. *…*.*and then feeling like I’m being left out deliberately because that’s how the bullying worked at the time*.”

[P21, female, White other, 36–45, urban]

Experiences such as bereavement or domestic violence were seen as relevant because they resulted in great deficits in emotional needs being met and then subsequent difficulties in forming warm and trusting relationships, both leading to loneliness.

#### 3.2 Current factors that maintain loneliness

Current factors influencing loneliness included separation and loss, difficulties in relationships, and physical barriers.

*3*.*2*.*1 Loss and separation*. Forms of loss that resulted in loneliness included romantic, platonic, physical and emotional loss, encapsulated in accounts of loss of friendships and of love, and loss through bereavement. One participant felt that their independence and well-being, especially their mental health, required them to stay physically away from family and friends, but that this nonetheless resulted in a sense of loneliness:

“*The one thing that I need for my sanity is my own space*…*although it’s better for me to live here* [in own home far from family and long-term friends] *because I have that*, *I do feel lonely*.”

[P2, female, 36–45, White British, urban]

For some, grief was a factor in feelings of loneliness and in being unable to engage in activities that might reduce these:

“*I have faced grieving for someone that died close to me*. *I despaired and just be at home alone and just not able to function…*”

[P48, female, 46–55, White other, urban].

*3*.*2*.*2 Compounding intersectional factors and external barriers*. For some participants a physical or age-related disability, was associated with barriers to using transport and getting involved in activities and this contributed to current feelings of loneliness, especially to the lack of a sense of belonging described under Theme 1.

“*I don’t think I have ever felt like not lonely*. *I think like disability*, *age*, *everything else creeps up on to make everything feel a hundred times worse*. *Loneliness to me is a disability*.”

[P52, female, 45–54, White Other, urban]

“*When I developed a physical disability*, *I found there was very little in my community as a person in my thirties that I was eligible to do*.”

[P44, female, White British, 36–45, urban]

Even within groups where they felt they belonged, people could experience inhibiting differences in attitudes, interests, speech style, access or social norms.

Other compounding intersectional factors cited by participants included sexuality, discrimination, race relations and the complexities of age differences in interactions with others.

*3*.*2*.*3 Time alone–impacts of solitude*. Some participants described negative effects of spending time physically alone, including for some a loss of skills needed to interact with others.

“*It will affect my mood*, *it will make me feel low*. *It will affect my loneliness greatly if there are several days I have not been out somewhere or seen someone or had face-to-face time with people*…”

[P23, male, Asian British, 36–45, urban]

“*You forget how to socialise*. *You become kind of quite selfish*, *actually*, *you don’t think about other people’s feelings*.”

[P42, female, Asian, 36–45, urban]

However, some people described a need to withdraw and ensure periods of solitude, even if this might exacerbate loneliness:

“*I need time on my own to recover and relax and rejuvenate but I don’t think it’s good to be on my own for long periods of time*.”

[P8, male, White British, 36–45, urban]

### Theme four: Ways of reducing loneliness

Two broad sub-themes emerged: external and internal approaches to reducing loneliness. These were categorised based on participants’ emphasis and experiences, but were often intertwined rather than being two clearly distinct categories. Strategies and ideas classed as “external ways to reduce loneliness” are those where participants emphasised how their experience depended on specific needs being met by people or environments external to themselves; those classed as “internal ways to reduce loneliness” highlight internal changes in thought patterns or other psychological shifts in participants’ ways of being and relating to the world around them.

Overall, different forms of social contact were seen as important for many participants in reducing loneliness, with quality of relationships and a sense of “belonging” being attributes of social contact that were particularly pertinent. Mental health challenges could act as a barrier to improving social contact and so addressing this was also seen as a key step for some participants. A booklet [31] (http://tiny.cc/Lonely) drafted by members of the research team summarises suggestions from study participants on coping with loneliness.

#### 4.1 External ways to reduce loneliness

Many participants described social contact as key to feeling less lonely. Some people had a preference for specific types of contact, whereas for others, any contact with people was important. Participants also described how volunteering gave them a sense of purpose and connection.

For some, just being able to get out, locally or in nature, was enough to lessen feelings of loneliness. Sometimes getting out resulted in unplanned encounters with people, as was the development of hobbies that could be enjoyed alone.

“*Try and communicate with people*. *Even if it’s going to [botanic] gardens by yourself*, *you’re going to meet people when you sit down at the café and they’re going to say to you* “*isn’t the weather lovely*?”

[P38, male, Black, 46–55, urban]

The quality of connection, and being able to talk about how you were feeling was essential for many:

“*My advice would be to talk to*…*someone that you trust*…*if you say it out loud*…*it does lift your feelings a bit because you’ve connected with someone*, *you’ve shared*, *you’ve*…*offloaded to them*.”

[P32, female, Asian, 26–35, urban]

Accessing mental health support was cited by some as a key first stage in decreasing loneliness by helping to improve mood and anxiety, and being able to reflect on oneself and develop better coping skills:

“*Before I*… *started medical treatment for my depression and anxiety I felt that being off feeling*. *Even if I was around people*, *I felt separated*, *that there was a kind of barrier that was stopping me from being able to communicate*.”

[P33, female, white British, 26–35, urban]

Taking part in activities and shared interests was mentioned by many participants, with “*structured socialisation*” being a route into developing new friendships or sense of belonging to a group, and technology being an enabler for some. Often, the shared interests gave purpose and fostered connection.

“*Structured socialisation*, *it provides that structure and*… *gives you a purpose so that you feel compelled to keep going and make friends and stuff and be sociable with other people*.”

[P5, female, White British, 26–35, rural]

“*The gym was really great*, *it was quite near me you know*, *and I’d never been to the gym before but I really got into that*, *and I was just getting to know people*.”

[P6, female, White other, 66–75, urban]

*‘‘I managed to find the live role-playing community*… *I have organised events to help kind of increase the hobby outreach and activities within the hobby*.”

[P19, female, White British, 36–45, urban]

For this participant, finding her kind of people online led to her then taking the initiative and organising her own online events and thus wrapping this community around her. Naturally occurring proximity, as well as freely chosen activities, could foster important connections: one participant found that living in a multiple occupancy house helped build friendships that increased their sense of belonging despite mental health struggles:

“*I had people that were close… in proximity quite a lot*, *and we got to become really good friends*, *and I felt safe in that*… *friendship and then was able to open up*, *even when I wasn’t feeling great*”

[P5, female, White British, 26–35, rural]

Thus finding a greater sense of belonging in a group or community could help people feel less lonely. Positive neighbourly encounters and informal interactions within a local community lessened feelings of loneliness for some. Others felt less lonely when connected to communities with whom they had something in common, such as people of the same ethnicity:

“*The ones that make me feel less lonely*, *…for a long time now*, *are my black friends*. *I feel more connected… I can express myself more freely without having to talk about certain things*… *so ethnicity is*… *playing a massive role in terms of feelings of loneliness*.”

[P37, female, 26–35, Black African, urban]

Religious worship, volunteering and acquiring a pet were among the other ways of connecting with others and becoming part of a valued group that were discussed:

“*Get a dog*, *if it’s possible because*, *you know*, *it’s much easier to interact with people in that case*.”

[P44, female, White British, 36–45, urban]

“*The* [volunteer job] *has been life changing*…*For me that connection*…*And people listening*, *realising that they are connecting*…*I feel like I’m doing something good*.”

[P9, female, White Other, 36–45, urban]

Some participants reflected that finding new activities and purpose required them to make a concerted effort:

“*I just have this thing that*, *when you walk around a town*…*there’s always posters and notices up of things happening…*, *even in your local* [supermarket]…*you’ll have what’s happening in your local community*…*libraries have loads of things happening now*.”

[P2, female, White British, 45–56, urban]

“*When I was* [living] *alone in a flat*… *I was much more lonely because I wasn’t tagged into those things which helped me to be less lonely*…*But we have to find these things and*, *if they make sense*, *they give us community*.”

[P13, male, White British, 66–75, urban]

#### 4.2 Internal ways to reduce loneliness

Internal strategies leading to better self-awareness, sometimes followed by taking steps to reducing anxiety and improving mood, were mentioned by many participants:

“*I’m more recognising it now in the last few years*…*what loneliness looks and feels like and therefore*, *because you can recognise it*, *you suddenly think oh I haven’t seen anybody for a while*, *I need to*…*make more of an effort now*.”

[P14, female, white British, 46–55, rural]

Participants reflected on relationships and learning to recognise what worked well for them and what made loneliness worse, thus improving the quality and connection in relationships:

“*With loneliness*, *what I can control is*… *who I’m around and who I feel most comfortable with*, *so maybe not hanging on to these friendships that make me feel even more lonely*, *even more isolated*.”

[P37, female, black, 26–35, urban]

Some people reported that acceptance of having time by yourself, being connected with yourself and your wishes and desires, was important. Carrying out activities like writing in a journal or art was helpful. For some, internal reflections and time alone led to better quality relationships and a reduction in loneliness.

“*To not beat yourself up*, *it’s not because there’s anything wrong with you*, *and that spending time on your own can be good ‘cause you can do things that you can then share with people*.”

[P2, female, White British, 45–56, urban]

Participants’ thoughts and feelings about isolation could also change because of psychological therapies, potentially resulting in taking steps to increase connection with others:

“*Through therapy and facing what I’ve been doing*…*in terms of anxiety or depression*, *I understand that*…*being sociable*, *going out and meeting people is healthy*, *and the longer I stay isolated… the harder it gets*”

[P26, male, White British, 36–45, urban]

## Discussion

### Main findings

Our findings conveyed how differently loneliness is experienced by different people, as expressed in terms of emotions, or even physical sensations. Loneliness appeared to comprise psychological elements, related to people’s thoughts and feelings about themselves in relation to other people and wider groups and communities, and social elements, related to the impacts of everyday interactions and contacts. Participants described a range of contributing factors to the origins of their loneliness, and the ways that their loneliness was maintained. They also perceived clear links between their loneliness and their mental health, and vice versa, and sometimes a feedback loop between the two.

Our participants’ accounts, as encapsulated in Theme 1, confirm that feelings of loneliness do not simply relate to dissatisfaction with the amount of time spent with others, but, perhaps more centrally, to not feeling connected to them in meaningful ways, and to not experiencing a sense of belonging. Loneliness has long been characterised as a physically and psychologically harmful manifestation of fundamental needs for social connection not being met [32]. Our findings regarding the importance of sense of belonging can be connected to investigations of loneliness from a social identity perspective, which supports that having multiple valued group memberships is associated with less loneliness and greater well-being [33]. The ways in which participants described both the nature of their loneliness and its origins were diverse, congruent with quantitative findings that suggest a complex causal web underlying loneliness [1]. Contributing factors identified included external factors such as losses and transitions or excessive time alone, but also aspects of personality such as introversion or lack of self-confidence, as well as the long-range impacts of trauma and adverse early experiences on the ability to form relationships (Theme 3).

Some of the themes illuminate aspects of loneliness relevant across populations, but a central aim of our work was to better understand loneliness among people living with mental health conditions. We found that loneliness and mental health appear intertwined in several ways (Themes 2). Participants described how feelings of not being connected to others could arise directly from a range of mental health conditions by pathways including feeling negative about self and others; withdrawing when depressed; and feeling unable to connect with others even when in company because of preoccupying social anxiety and hearing inner voices with negative content impeding trust and ability to socialise. This is in keeping with findings of greater loneliness associated with a range of mental health conditions [13, 34–36]. Some participants also described how their loneliness could lead directly to onset or exacerbation of the mental health conditions they were experiencing including depression, which is congruent with longitudinal studies establishing loneliness as an independent risk factor for depression [11, 12], and with findings of a bidirectional relationship between loneliness and depression [37].

Going beyond direct links from mental health symptoms to loneliness, the actions people took to cope with their mental health problems sometimes also placed them at greater risk of loneliness, such as when they withdrew from the stresses of social contacts and activities in order to recover and to ensure they had time and energy to cope with pressing practical needs. The circular relationship between loneliness and mental ill health identified in Theme 3 evoked the paradox identified by Achterbergh et al. [21] in their meta-synthesis of qualitative studies on loneliness and depression among young people: social withdrawal to cope with mental ill health can result in loneliness that further exacerbates mental health problems.

Significant contributors to loneliness among people with mental health problems in our sample were stigma and self-stigma associated with mental health problems, especially as impediments to having a sense of belonging. This is in keeping with previous findings of an association between self-stigma and social withdrawal among people with severe mental health problems [38], and of high levels of persisting mental health stigma despite a longstanding UK public anti-stigma campaign, focused primarily on common mental health problems [39].

Experiences of stigma and social exclusion related to mental health intersected for many participants with social exclusion associated with being part of other disadvantaged or marginalised groups at increased risk of mental health problems, including racial and sexual minorities and people with disabilities, and with impacts of social deprivation. As Lever-Taylor et al. [40] argue in their qualitative study of perinatal women, an intersectional focus is helpful in understanding social drivers of loneliness. Participants’ accounts of the many impediments to a sense of belonging, including stigma, reinforce a need to take a societal and community as well as individual approach to understanding loneliness. Thus, loneliness appears to result not only from individuals’ inability to connect, but also from failures of communities to welcome and integrate people living with mental health problems, and from practical barriers to connecting with others that result from poverty [41].

Many of the themes and sub-themes so far discussed cohere with previous literature, for example illuminating potential mechanisms underpinning epidemiological findings. To our knowledge, our exploration of the strategies and types of help people employ to combat loneliness is novel (Theme 4). We found that many participants were aware of their loneliness and its impacts, and of a variety of strategies that could help: we reflect further on implications for interventions below.

### Strengths and limitations

Our study represents a substantial contribution to the limited qualitative literature on loneliness among people with mental health problems. We recruited a diverse sample, encompassing a range of backgrounds, types of mental health problem, service use histories and locations. Although using a digital platform for most interviews will have excluded a substantial section of the population using mental health services, we did conduct some interviews by telephone and face-to-face to accommodate the digitally excluded. Lived experience was embedded in the study at each stage, with people with relevant personal experiences designing interview guides, conducting interviews, analysing data and writing up findings.

Limitations include that we conducted a broad-brush analysis of a large sample of interviews, and searched for commonalities across a group that was very diverse in characteristics and experiences rather than focusing in more depth on more defined groups. Some people discussed links between specific mental health conditions and onset or exacerbation of loneliness, but our sample was very varied and diagnosis was based on self-report, so we cannot discuss links between particular symptoms and conditions and loneliness in depth; a potential direction for future work. We did not measure severity of mental health difficulties, and note that while a majority had used specialist mental health services (inpatient or community teams), thirteen participants reported not having used services for mental health.

The majority of interviews were conducted during the COVID-19 pandemic, and we have reported baseline [23] and follow-up [26] findings elsewhere, but the pandemic context may have influenced accounts of loneliness, even though participants were encouraged to talk about experiences of loneliness in general rather than specifically in a pandemic context, and appeared for the most part to be doing so.

### Research and clinical implications

The rich and complex accounts given by participants of the nature of their experiences of loneliness, and the great variety of pathways into and out of it, indicate considerable further scope for research to understand associations between loneliness and mental health. In qualitative research, many of our themes warrant more in-depth exploration, including focusing on particular groups at high risk of loneliness, or who are currently lonely. In quantitative research, it would be valuable to investigate further the longitudinal relationships between loneliness and mental health problems, and the extent to which contributory factors identified in our study are also reflected in epidemiological analyses. Much research on loneliness has employed single-item or brief measures not tailored to people with mental health conditions: we reflect that such measures are unlikely to capture the diversity of experiences of loneliness and links to mental health.

As yet there are very few clearly evidence-based strategies for helping with loneliness among people living with mental health problems [16]. Our findings show people with mental health problems recognising and taking steps to address their loneliness. This supports the greater deployment of co-production approaches, incorporating the forms of help that people with relevant personal experience see as potentially effective. With few exceptions [42], interventions tested thus far have tended not to be co-produced. The diversity of pathways into and out of loneliness that people described, and of suggested strategies, indicate that a variety of approaches are potentially helpful depending on the nature and context of loneliness, delivered singly or in combination. These would include self-help and psychoeducation about day-to-day strategies that people have found helpful, psychological interventions focused on thoughts and feelings about others, and social approaches to help people develop meaningful connections and a sense of belonging [15, 43].

The range of mental health-related factors triggering and perpetuating loneliness suggest benefits to developing or adapting specific strategies for this population rather than deploying strategies developed for the general population. Approaches to loneliness in a mental health context based on peer support seem rarely to have been reported, but have clear potential benefits such as in overcoming obstacles due to stigma and self-stigma, fostering a sense of belonging and supporting people with self-help strategies to reduce loneliness. Finally, our perspective in this study was on improving the support offered in mental healthcare settings, but our findings illustrated the community-level, intersectional and socio-economic drivers of loneliness in multiple ways. We suggest that addressing such drivers will have a central influence on whether levels of loneliness can be reduced among people living with mental health problems.

### Lived experience commentary written by Beverley Chipp

This paper must be understood in the context of the COVID-19 lockdown occurring when we had only conducted a few interviews. All participants had already felt lonely as this was our inclusion criteria, but limitations upon movement and social contact imposed by government, and, significantly, the cessation of many support services will have influenced subsequent interviewees’ responses. For some these changes made things worse and for others life became easier. The shifts triggered significant reflections on life and participants’ relationships, and perhaps provided richer data than would have been collected in normal times.

Access to mental health support has been increasingly difficult over the last decade but the suspension of face to face services, or for some, services altogether, exacerbated loneliness leaving many feeling brushed aside.

We learned that loneliness and social isolation were distinct entities and experienced in multiple and diverse ways. The causes and perpetuating factors were more complex than we had imagined, even though we identified with some of the narratives.

Solutions need to take a psychologically informed, holistic approach rooted in community and co-production. Without addressing stigma and other barriers within society–prejudice and discrimination, hostility against those that don’t ‘fit’–people’s sense of belonging will be hindered, and loneliness increased. Where loneliness has childhood origins it may be more difficult to unpick. Simply engineering people to be with other people is too simple a solution for most individuals also living with mental health conditions.

Commonality between lived experience researchers and participants fostered greater trust in interviews and thus sharing of deeper insights. We felt that research often stays within a ‘research bubble’, not reaching the people it directly affects. Recognising the wealth garnered from asking participants ‘what helped?’ and their willingness to offer suggestions, we co-produced a self-help booklet (http://tiny.cc/Lonely) which acknowledges their resourcefulness.

## Supporting information

S1 Appendix Interview topic guide. (DOCX)

S2 Appendix Revised interview topic guide. (DOCX)

## References

[1] Lim MH, Eres R, Vasan S. Understanding loneliness in the twenty-first century: an update on correlates, risk factors, and potential solutions. Social Psychiatry and Psychiatric Epidemiology 2020; 55, 793–810. doi: 10.1007/s00127-020-01889-7 32524169

[2] Perlman D, & Peplau L. A. Toward a Social Psychology of Loneliness. In Gilmour R., & Duck S. (Eds.), Personal Relationships: 3. Relationships in Disorder (pp. 31–56). London: Academic Press. (1981).

[3] Lauder W, Sharkey S, Mummery K. A community survey of loneliness. Journal of Advanced Nursing 2004;46(1):88–94. doi: 10.1111/j.1365-2648.2003.02968.x 15030445

[4] Badcock JC, Shah S, Mackinnon A, Stain HJ, Galletly C, Jablensky A, et al. Loneliness in psychotic disorders and its association with cognitive function and symptom profile. Schizophr Res 2015;169 (1–3):268–273. doi: 10.1016/j.schres.2015.10.027 26527247

[5] Petitte T, Mallow J, Barnes E, Petrone A, Barr T, Theeke L. A systematic review of loneliness and common chronic physical conditions in adults. The Open Psychology Journal 2015, 8(Suppl 2), p113. doi: 10.2174/1874350101508010113 26550060 PMC4636039

[6] Hawkley LC, Thisted RA, Masi CM, Cacioppo JT. Loneliness predicts increased blood pressure: 5-year cross-lagged analyses in middle-aged and older adults. Psychology and Aging 2010; 25(1), p 132. doi: 10.1037/a0017805 20230134 PMC2841310

[7] Steptoe A, Shankar A, Demakakos P, Wardle J. Social isolation, loneliness, and all-cause mortality in older men and women. Proceedings of the National Academy of Sciences of the United States of America 2013, 110(15), pp 5797–801. doi: 10.1073/pnas.1219686110 23530191 PMC3625264

[8] Valtorta N, Kanaan M, Gilbody S, Ronzi S, Hanratty B. Loneliness and social isolation as risk factors for coronary heart disease and stroke: systematic review and meta-analysis of longitudinal observational studies. Heart 2016, 102:1009–1016. doi: 10.1136/heartjnl-2015-308790 27091846 PMC4941172

[9] Gerst-Emerson K, Jayawardhana J. Loneliness as a Public Health Issue: The Impact of Loneliness on Health Care Utilization Among Older Adults. American Journal of Public Health, 2015;105(5), pp 1013–1019. doi: 10.2105/AJPH.2014.302427 25790413 PMC4386514

[10] Newall N, McArthur J, and Menec VH. A Longitudinal Examination of Social Participation, Loneliness, and Use of Physician and Hospital Services. Journal of Aging and Health 2015 27(3), pp 500–518. doi: 10.1177/0898264314552420 25288587

[11] Mann F., Wang J., Pearce E., Ma R., Schlief M., Lloyd-Evans B. et al. Loneliness and the onset of new mental health problems in the general population. Soc Psychiatry Psychiatr Epidemiol (2022). doi: 10.1007/s00127-022-02261-7 35583561 PMC9636084

[12] Lee SL, Pearce E, Ajnakina O, Johnson S, Lewis G, Mann F et al. The association between loneliness and depressive symptoms among adults aged 50 years and older: a 12-year population-based cohort study. Lancet Psychiatry 2021; 8: 48–57. doi: 10.1016/S2215-0366(20)30383-7 33181096 PMC8009277

[13] Wang J, Mann F, Lloyd-Evans B, Ma R, Johnson S. Associations between loneliness and perceived social support and outcomes of mental health problems: a systematic review. BMC Psychiatry 2018 May 29;18(1):156. doi: 10.1186/s12888-018-1736-5 29843662 PMC5975705

[14] Ma R, Wang J, Lloyd-Evans B, Marston L, Johnson S. Trajectories of loneliness and objective social isolation and associations between persistent loneliness and self-reported personal recovery in a cohort of secondary mental health service users in the UK. BMC Psychiatry 2021 21 421. doi: 10.1186/s12888-021-03430-9 34425767 PMC8381487

[15] Mann F, Bone JK, Lloyd-Evans B, Frerichs J, Pinfold V, Ma R et al. A life less lonely: the state of the art in interventions to reduce loneliness in people with mental health problems. Social Psychiatry and Psychiatric Epidemiology 2017; Jun;52(6):627–638. doi: 10.1007/s00127-017-1392-y 28528389 PMC5487590

[16] Ma R, Mann F, Wang J, Lloyd-Evans B, Terhune J, Al-Shihabi A et al. The effectiveness of interventions for reducing subjective and objective social isolation among people with mental health problems: a systematic review Social Psychiatry and Psychiatric Epidemiology 2020; 55, 839–876. doi: 10.1007/s00127-019-01800-z 31741017 PMC7303071

[17] Svendsen L. A Philosophy of Loneliness. 2017; Reaktion Books.

[18] Alberti FB. A Biography of Loneliness: The History of an Emotion. 2021; OUP Oxford.

[19] Sagan O. The loneliness of personality disorder: a phenomenological study. Mental Health and Social Inclusion. 2017; 21(4), pp. 213–221.

[20] Lindgren BM, Sunbaum J, Eriksson M, Graneheim UH. Looking at the world through a frosted window: experiences of loneliness among persons with mental ill-health Journal of Psychiatric and Mental Health Nursing 2014 21, 114–120. doi: 10.1111/jpm.12053 23530616

[21] Achterbergh L, Pitman A, Birken M, Pearce E, Sno H, Johnson S. The experience of loneliness among young people with depression: A qualitative meta-synthesis of the literature. BMC Psychiatry; 2020; 20(1). doi: 10.1186/s12888-020-02818-3 32831064 PMC7444250

[22] Pitman A, Mann F, Johnson S. Advancing our understanding of loneliness and mental health problems in young people. The Lancet Psychiatry 2018; 5, 12, 955–956. doi: 10.1016/S2215-0366(18)30436-X 30477651

[23] Gillard S, Dare C, Hardy J, Nyikavaranda P, Rowan Olive R, Shah P, et al. Experiences of living with mental health problems during the COVID-19 pandemic in the UK: a coproduced, participatory qualitative interview study. Social Psychiatry and Psychiatric Epidemiology 2021 56, 1447–1457. doi: 10.1007/s00127-021-02051-7 33665680 PMC7931976

[24] King C and Gillard S. Bringing together coproduction and community participatory research approaches: Using first person reflective narrative to explore coproduction and community involvement in mental health research Health Expectations (2019) 22: 701–708. doi: 10.1111/hex.12908 31187556 PMC6737774

[25] Vera San Juan N, Shah P, Schlief M, Appleton R, Nyikavaranda P, Birken M et al. Service user experiences and views regarding telemental health during the COVID-19 pandemic: A co-produced framework analysis PLOS ONE 2021; 16(9): e0257270. doi: 10.1371/journal.pone.0257270 34529705 PMC8445423

[26] Shah P, Hardy J, Birken M, Foye U, Rowan Olive R, Nyikavaranda P et al. What has changed in the experiences of people with mental health problems during the COVID-19 pandemic: a coproduced, qualitative interview study Social Psychiatry and Psychiatric Epidemiology (2022) 57, 1291–1303. doi: 10.1007/s00127-022-02254-6 35267053 PMC8908744

[27] Zoom Video Communications, Inc. (2020). ZOOM cloud meetings (Version 4.6.9) [Mobile app]. App Store. https://apps.apple.com/us/app/zoom-cloud-meetings/id546505307https://apps.apple.com/us/app/zoom-cloud-meetings/id546505307

[28] King, N., and J. M. Brooks. 2017. Template Analysis for Business and Management Students. London: Sage.

[29] Braun V and Clark V, Reflecting on reflexive thematic analysis Qualitative Research in Sport, Exercise and Health, 2019;11:4, 589–597, doi: 10.1080/2159676X.2019.1628806

[30] QSR International Pty Ltd. NVivo (Version 12), 2018, https://www.qsrinternational.com/nvivo-qualitative-data-analysis-software/home

[31] Loneliness and social Isolation in Mental Health Research Network and NIHR Mental Health Policy Research Unit, Conversations Around Loneliness and Mental Health 2022 https://www.ucl.ac.uk/psychiatry/sites/psychiatry/files/conversations_around_lonelines_mental_health_18.01.2022.pdf

[32] Cacioppo JT, Cacioppo S. The phenotype of loneliness. European Journal of Developmental Psychology 2012; 9, 4, 446–452. doi: 10.1080/17405629.2012.690510 23024688 PMC3459997

[33] Haslam C, Jetten J, Cruwys T, Dingle J, Haslam A. The New Psychology of Health Unlocking the Social Cure 2018; Routledge.

[34] Erzen E, Çikrikci Ö. The effect of loneliness on depression: A meta-analysis. International Journal of Social Psychiatry 2018; 64(5):427–435. doi: 10.1177/0020764018776349 29792097

[35] Chau AK, Zhu C, & So SHW. Loneliness and the psychosis continuum: a meta-analysis on positive psychotic experiences and a meta-analysis on negative psychotic experiences. International Review of Psychiatry 2019; 31(5–6), 471–490. doi: 10.1080/09540261.2019.1636005 31331209

[36] Loades ME, Chatburn E, Higson-Sweeney N, Reynolds S, Shafran R, Brigden A, et al. Rapid Systematic Review: The Impact of Social Isolation and Loneliness on the Mental Health of Children and Adolescents in the Context of COVID-19. Journal of the American Academy of Child & Adolescent Psychiatry 2020; 59(11):1218–1239. doi: 10.1016/j.jaac.2020.05.009 32504808 PMC7267797

[37] Nuyen J, Tuithof M, De Graff R, Van Dorsselaer S, Kleinjan M, Ten Have M. The bidirectional relationship between loneliness and common mental disorders in adults: findings from a longitudinal population-based cohort study Social Psychiatry and Psychiatric Epidemiology. 2020; 55, 1297–1310. doi: 10.1007/s00127-019-01778-8 31538206

[38] Abiri S, Oakley LD, Hitchcock ME, Hall A. Stigma Related Avoidance in People Living with Severe Mental Illness (SMI): Findings of an Integrative Review. Community Ment Health J 2016; 52(3):251–61. doi: 10.1007/s10597-015-9957-2 26668008

[39] Henderson C, Gronholm PC. Mental Health Related Stigma as a ‘Wicked Problem’: The Need to Address Stigma and Consider the Consequences. International Journal of Environmental Research and Public Health 2018; 15, 1158; doi: 10.3390/ijerph15061158 29865225 PMC6024896

[40] Lever-Taylor B, Howard LM, Jackson K, Johnson S, Mantovani N, Nath S et al. Mums Alone: Exploring the Role of Isolation and Loneliness in the Narratives of Women Diagnosed with Perinatal Depression J Clin Med 2021; 24;10(11):2271. doi: 10.3390/jcm10112271 34073903 PMC8197355

[41] Macdonald SJ, Nixon J, Deacon L. ‘Loneliness in the city’: examining socio-economics, loneliness and poor health in the North East of England Public Health 2018; 165, 88–94. doi: 10.1016/j.puhe.2018.09.003 30384033

[42] Lloyd-Evans B, Frerichs J, Stefanidou T, Bone J, Lewis G, Billings J et al. The Community Navigator Study: Results from a feasibility randomised controlled trial of a programme to reduce loneliness for people with complex anxiety or depression PLOS ONE 2020; doi: 10.1371/journal.pone.0233535 32469922 PMC7259554

[43] Pearce E, Myles-Hooton P, Johnson S, Hards E, Olsen S, Clisu D et al. Loneliness as an active ingredient in preventing or alleviating youth anxiety and depression: a critical interpretative synthesis incorporating principles from rapid realist reviews Translational Psychiatry 2021 11:628. doi: 10.1038/s41398-021-01740-w 34893578 PMC8661314

