# Supplementary material for: Exploring the experiences of loneliness in adults with mental health problems: A participatory qualitative interview study
Source: PLoS One. 2023 Mar 7;18(3):e0280946. doi: 10.1371/journal.pone.0280946 (PMC9990944; doi:10.1371/journal.pone.0280946)
Supplement: S1 Appendix — (DOCX) [file pone.0280946.s001.docx]

**Appendix 1**

**Interview Topic Guide**

**Title of Study: Exploring the experiences of loneliness with people living with mental health problems**

1. First of all, could you tell us about who you have contact with, both face-to-face and via technology?

Interviewer Prompts:

• For example, are there people you see each day, every week or on a regular basis?

• Do you belong to any groups or clubs? Do you work?

• Could you tell me about your use of technology and social media (Facebook, Instagram, Twitter, WhatsApp, Skype, apps, online forums, assistive tech etc)?

Experience of loneliness

2. Can you tell me what the word lonely means to you? How would you define it?

Interviewer Prompts:

• How does it make you feel? Physically, psychologically, emotionally e.g. social anxiety.

3. Has there been a time or times in your life when you have been lonely?

4. Would you say you feel lonely in your life in general at the moment?

What is/was that like?

Interviewer Prompts:

• How does loneliness impact on you day to day?

• Are there things that you think have triggered or underlie your feeling of loneliness? E.g. age, culture, personality characteristics, difficulty fitting in, stopping work, moving location, loss of a partner or family.

• Are there situations or times when you feel more lonely than others? Prompt: e.g. seasonal, not working.

Social contact and loneliness

5. Do you feel lonely when you are in the company of others?

Are there any kinds of social contact that make you feel more or less lonely?

Interviewer Prompts:

• E.g. groups, family, special friend, in person rather than technology.

• When you meet people, do you feel that you can talk to them easily?

6. What is the difference between meeting people, making friends and maintaining friendships?

7. Do you think that spending time on your own can be helpful/therapeutic?

8. Does spending too much time on your own become unhealthy?

• [If yes] - How much is too much? Where is the line?

Loneliness and mental health

9. Do you think feeling lonely is connected to your mental health?

• [if yes]: In what ways?

Interviewer Prompts:

• Does feeling lonely make your mental health worse? In what ways?

• Do you think your mental health problems contribute to your loneliness? [Prompts: e.g. side effects of medication, the age you developed mental health problems.]

• Do you feel that loneliness is different/distinct from your mental health problems?

• Thinking back to when your mental health problems first started, do you think that feeling lonely came before or after?

• [If loneliness came first]: Has your experience of loneliness changed since having mental health problems?

10. Do you feel you belong in the community around you and with your family and friends?

• [If not]: Can you tell me more about this feeling of not belonging? In what ways, if any, does that relate to your feeling of loneliness?

11. Have you ever discussed feeling lonely with family, friends or any healthcare professional?

If so, what happened next, did it help?

Final questions:

12. Are there ways in which you have tried to reduce your loneliness? What has and has not worked?

Interviewer Prompts:

• Do you have coping strategies that you use?

• Have you had support with this? What kind of support? Has this helped?

13. What would not being lonely look like for you?

Interviewer Prompts:

• How do you imagine your life would be different?
