# Supplementary material for: Exploring the experiences of loneliness in adults with mental health problems: A participatory qualitative interview study
Source: PLoS One. 2023 Mar 7;18(3):e0280946. doi: 10.1371/journal.pone.0280946 (PMC9990944; doi:10.1371/journal.pone.0280946)
Supplement: S2 Appendix — (DOCX) [file pone.0280946.s002.docx]

**Appendix 2**

**Revised Interview Topic Guide**

Title of Study: Exploring the lived experiences of loneliness and isolation with people with mental health problems during the COVID-19 pandemic in the UK.

The first five questions focused on the experiences of people during the COVID-19 pandemic and are excluded from the topic guide presented here, as the findings are not reported in this paper.

Experience of loneliness

6. Can you tell me what the word lonely means to you? How would you define it?

Prompts: How does it make you feel? Physically, psychologically, emotionally e.g. social anxiety.

7. Has there been a time or times in your life when you have been lonely?

Would you say you feel lonely in your life in general at the moment?

What is/was that like?

Prompts: Are there things that you think have triggered or underlie your feeling of loneliness? E.g. age, culture, personality style, , difficulty fitting in, stopping work, moving location, loss of a partner or family.

Are there situations or times when you feel more lonely than others? Prompt: e.g., seasonal, not working.

Social contact and loneliness

8. Do you feel lonely when you are in the company of others?

Are there any kinds of social contact that make you feel more or less lonely?

Prompts: E.g., groups, family, special friend, in person rather than online or using technology. When you meet people in person, do you feel that you can talk to them easily?

9. Do you think that spending time on your own can be helpful/therapeutic?

10. Does spending too much time on your own negatively affects your wellbeing?

• [If yes] - How much is too much? Where is the line?

Loneliness and mental health

11. Do you think feeling lonely is connected to your mental health?

• [if yes]: In what ways?

Prompts: Does feeling lonely make your mental health worse? In what ways? Do you think your mental health problems or your treatment contribute to your loneliness? [Prompts: e.g. side effects of medication, the age you developed mental health problems.]

• Thinking back to when your mental health problems first started, do you think that feeling lonely came before or after?

• [If loneliness came first]: Has your experience of loneliness changed since having mental health problems?

12. Do you feel you belong in the community around you?

Prompts: explore different communities: neighbourhood, family and friendship groups, communities of interest [If not]: Can you tell me more about this feeling of not belonging? In what ways, if any, does that relate to your feeling of loneliness?

Final questions:

13. Are there ways in which you have tried to reduce your loneliness? What has and has not worked?

Prompts: Do you have ways of coping? Have you had any support? What kind of support? Has this helped?

14. What would not being lonely look like for you?

Prompts: How do you imagine your life would be different?
